# Visceral symptoms as a key diagnostic sign for the early infantile form of Niemann–Pick disease type C in a Russian patient: a case report

**DOI:** 10.1186/s13256-016-0925-4

**Published:** 2016-06-01

**Authors:** A. V. Degtyareva, S. V. Mikhailova, E. Y. Zakharova, E. L. Tumanova, A. A. Puchkova

**Affiliations:** 1 Federal State Budget Institution, Research Center for Obstetrics, Gynecology and Perinatology, Federal State Budget Institution, 117997 Oparina str. 4, Moscow, Russia; 2 Russian Children’s Hospital, 117997 Leninsky Prospect 117, Moscow, Russia; 3 State Institution Medical Genetic Research Center, 115478, Moskvorechje str. 1, Moscow, Russia; 4 Russian National Research Medical University, 117997, Ostrovitjanova str. 1, Moscow, Russia

**Keywords:** Niemann–Pick disease type C, Splenomegaly, Cholestasis, Miglustat

## Abstract

**Background:**

Niemann–Pick disease type C is a rare metabolic disease characterized by progressive neurological deterioration with childhood onset, and often results in premature mortality. Niemann–Pick disease type C has an extremely heterogeneous clinical presentation with a wide range of visceral and neurological signs and symptoms that are not specific to the disease, and which progress over varied periods of time. The incidence and epidemiology of Niemann–Pick disease type C in Russia have not been characterized. We report the case of a Russian newborn with early-infantile onset Niemann–Pick disease type C who displayed prolonged neonatal jaundice and hepatosplenomegaly.

**Case presentation:**

A 5-year-old white boy born to non-consanguineous Russian parents was originally diagnosed with galactosemia at the age of 2 months based on a raised blood galactose level. A galactose-free and lactose-free diet resulted in achievement of a normal galactose level, but hepatosplenomegaly and cholestatic signs persisted. Liver biopsy results hinted at possible Niemann–Pick disease type C, but differential diagnostic investigations for progressive familial intrahepatic cholestasis type 2 (Byler syndrome) indicated a heterozygous genotype suggestive of this disease. Further, progressive neurological symptoms prompted additional genetic analyses for possible Niemann–Pick disease type C, from which an as-yet unreported combination of known *NPC1* gene mutations was identified, and a final diagnosis of Niemann–Pick disease type C was established. The patient subsequently developed typical neurological symptoms of early-infantile Niemann–Pick disease type C, including vertical supranuclear ophthalmoparesis and cerebellar ataxia. Miglustat therapy was initiated 2.5 years ago, and some improvements in movement and speech have since been observed.

**Conclusions:**

This case illustrates the continued challenges associated with diagnosing Niemann–Pick disease type C based on the appearance of nonspecific cholestatic symptoms. Based on this case we recommend examination of all newborns and children who display unexplained cholestasis or isolated splenomegaly/hepatosplenomegaly during the first months of life for other signs of possible Niemann–Pick disease type C.

## Background

Niemann–Pick disease type C (NP-C) is a rare lysosomal storage disease in which impaired intracellular lipid trafficking leads to excess storage of cholesterol, sphingomyelin, glycosphingolipids, and sphingosine in the brain, liver, spleen, lungs, and other tissues [1]. The incidence of NP-C in Russia has not yet been established; only single case reports have been published to date [2].

**Fig. 1:**
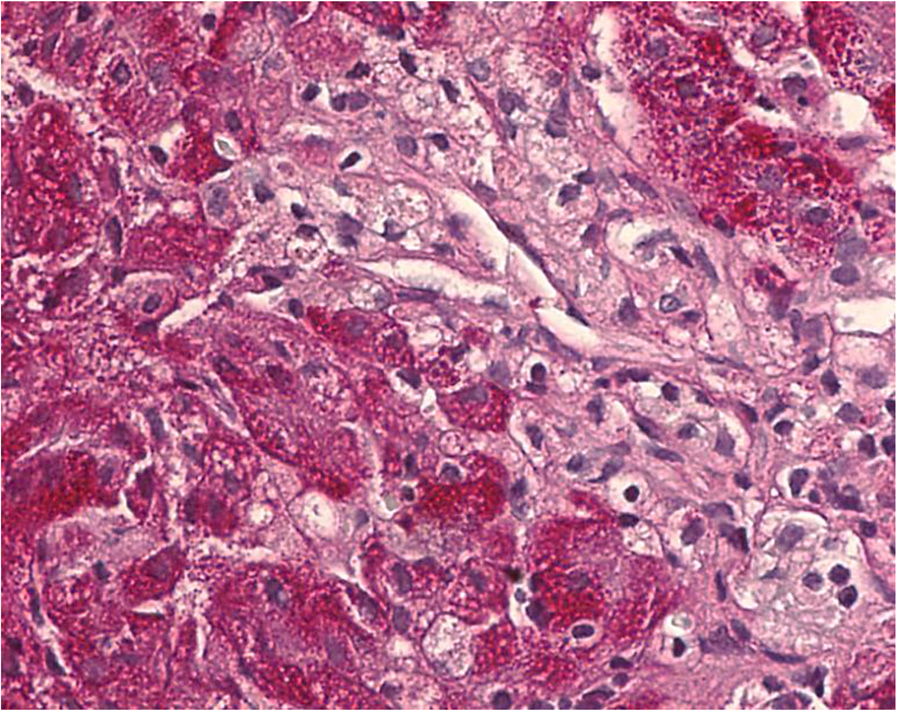
Histopathological liver biopsy findings. Hematoxylin and eosin staining with magnification ×400 showing swollen Kupffer cells with foamy cytoplasm, typical for Niemann–Pick disease type C

NP-C has an extremely heterogeneous clinical presentation characterized by a wide range of symptoms that are not specific to the disease, and which arise and progress over varied periods of time [1, 3]. Disease onset occurs in the neonatal period in a notable proportion of patients. In particular, NP-C represents one of the leading metabolic causes of cholestatic syndrome in babies [3]. Non-immune hydrops, ascites, and fetal hepatosplenomegaly are also often described in affected children [3]. Cholestasis and severe splenomegaly in the absence of portal hypertension are considered to be pathognomonic [4].

We report the case of a 5-year-old boy with early infantile onset NP-C that illustrates some of the challenges associated with diagnosing the disease early on in life based on the appearance of nonspecific cholestatic symptoms.

## Case presentation

The patient was a 5-year-old Caucasian boy (weight at birth 2900 g, height at birth 50 cm) born to non-consanguineous parents with no previous medical history of NP-C in Krasnodar, Russia. Jaundice was observed at the age of 3 days and was considered physiological. The child was therefore discharged. However, he was later hospitalized at a child care facility due to continued jaundice and mild hepatosplenomegaly (liver 2.5 cm and spleen 1 cm), at which point raised total and conjugated bilirubin, alkaline phosphatase, cholesterol, alanine aminotransferase (ALT), and aspartate aminotransferase (AST) were observed.

The results of his hematology and urine analysis tests were normal, but heightened nervous reflex irritability was observed. Blood polymerase chain reaction (PCR) testing for cytomegalovirus (CMV), herpes simplex virus (HSV), toxoplasmosis, and chlamydia infection were negative. However, a blood galactose of 12 ng/ml (upper limit of normal, 7.1 ng/ml) was observed, which increased to 20 ng/ml at repeat examination. On this basis, galactosemia was suspected and a galactose-free and lactose-free diet was prescribed. By the age of 2 months the patient’s blood galactose level was normalized, but cholestasis and hepatosplenomegaly persisted. He was therefore referred to the Research Center for Obstetrics, Gynecology and Perinatology at the Federal State Budget Institution in Moscow, Russia for further work up.

We observed hypotrophy and continued jaundice at our initial examination: body weight 4100 g (percentile 5 to 10); height 57 cm (percentile 25). Ongoing hepatosplenomegaly, cholestasis, and cytolysis were also confirmed, but synthetic liver function tests and standard metabolic parameters were all normal (Table 1). There were no signs of portal hypertension in spite of the enlarged spleen (+6.5 cm under the costal rib). His plasma chitotriosidase was slightly increased (251.5 nmol/mg/hour, normal range 4.5 to 198 nmol/mg/hour). Galactosemia was eventually ruled out based on normal galactose-1-phosphate-uridyl transferase enzyme levels and genetic testing. Differential diagnoses for an extensive group of other metabolic and non-metabolic diseases were eliminated: biliary atresia, Gaucher disease type 1 (GD1), α-1-antitrypsin deficiency, tyrosinemia, citrullinemia type 2, progressive familial intrahepatic cholestasis type 3, mitochondrial disorders, Alagille syndrome, and others.

**Table 1:** Blood biochemical analysis at the age of 2 months and 1 week

Parameters	Value	Normal range
Total bilirubin, mkM/l	223	3.4–21.0
Conjugated bilirubin, mkM/l	117	0–5.5
Gamma glutamine transferase, U/l	73	0–50
Alkaline phosphatase, U/l	730	50–360
Alanine aminotransferase, U/l	313	0–40
Aspartate aminotransferase, U/l	673	0–40
Creatine phosphokinase, U/l	167	0–171
Albumin, g/l	45	35–50
Cholinesterase, U/l	6237	3930–10800
Fibrinogen, g/l	3,9	2–4
Prothrombin time index, %	94	80–120
Lactate, mmol/l	1.1	1.2
Glucose, mmol/l	3.7	3.5–6.4

A liver biopsy revealed swollen Kupffer cells with foamy cytoplasm, which are considered typical histopathological hallmarks of NP-C (Fig. 1). A preliminary diagnosis of NP-C was therefore concluded. No causal *NPC2* gene mutations were detected; while parallel investigations for progressive familial intrahepatic cholestasis type 2 (Byler syndrome) indicated a heterozygous genotype suggestive of this disease.

Typical symptoms of Byler syndrome include progressive cholestasis with low blood gamma glutamyltransferase (GGT) levels and severe pruritus. Liver transplantation is always indicated for Byler syndrome because of its association with biliary cirrhosis and poor quality of life. During the examination of our patient at the age of 2 to 3 months he had slightly increased (near-normal) GGT and he was too young for pruritus. Pruritus usually appears at the age of 4 to 5 months or later, which is why we could not exclude Byler syndrome. On the other hand we could not insist on the performance of genetic testing for *NPC1* in this case because of the expense involved and the fact that his family was not ready.

The patient undertook a high medium-chain triglyceride diet with fat-soluble vitamins, and ursodeoxycholic acid therapy. Jaundice eventually disappeared by 5 months of age, and by the age of 2 years his hepatomegaly had resolved. His splenomegaly had also decreased (6 to 7 cm). Later on it became obvious that he did not have Byler syndrome as his cholestasis resolved, he did not have pruritus, and he had splenomegaly without portal hypertension. He was therefore not indicated for liver transplantation. On balance, we decided to suspend further genetic testing.

After a bout of acute otitis he stopped walking on his own aged 2 years and 10 months. He also stopped eating independently due to a pronounced tremor. Brain MRI findings were normal. These neurological symptoms prompted further diagnostic tests for possible NP-C, and complete *NPC1* gene mutation analysis identified an as yet unreported combination of known mutations c.2777C>T (p.Ala926Val: CM077214) and c.2196_2197insT.

Retrospective analysis of his neurological status showed normal psychomotor development during his first year of life, but he did display minor deficits (for example, inward turning of his feet, awkward gait) that were probably the first neurological disease manifestations. An observable tremor first appeared when he was 2.5-years old.

At the time of reporting he is 5-years old and displays abnormal saccadic eye movements, trunk ataxia, hypotonia, subcortical cerebellar dysarthria and dysmetria, and intention tremor. He continues to walk with assistance but his gait is ataxic-polyneuropathic. He has also lost 2 kg in body weight since the initial onset of neurological manifestations, and displays signs of first-degree hypotrophy. His splenomegaly persists (spleen size, 7 cm).

Miglustat (Zavesca®; Actelion Pharmaceuticals Ltd) was initiated 2.5 years ago with appropriate dietary alterations. Positive changes have since been observed, including reduced tremor and decreased muscle hypotonia. The patient can sometimes walk without assistance and he exhibits a degree of improvement in his speech. He has also gained weight. His liver function test results remain normal, and splenomegaly has persisted without signs of portal hypertension.

## Discussion

This case report is based on a typical case of early-infantile onset NP-C, and illustrates some of the challenges associated with diagnosing patients with NP-C early on in life based on the appearance of nonspecific cholestatic symptoms. Despite the appearance of neonatal cholestasis, which prompted investigations for possible Gaucher disease (GD) or galactosemia, neurological signs suggestive of NP-C only became apparent at the end of the first year of life [1, 3].

Cholestasis and splenomegaly were the first clinical signs of NP-C in our patient, and NP-C ranks highly among possible metabolic causes of neonatal cholestasis [3]. Nevertheless, a differential diagnosis was performed in a way that took into account an extensive group of other metabolic and non-metabolic diseases, including: GD, galactosemia, α-1-antitrypsin deficiency, tyrosinemia, citrullinemia type 2, progressive familial intrahepatic cholestasis type 3, mitochondrial disorders, Alagille syndrome, and others.

Swollen Kupffer cells with foamy cytoplasm in liver biopsy specimens was the first strong sign of NP-C, but even these histopathological changes are not specific to the disease [4]. Filipin staining in cultured skin fibroblasts is considered a key diagnostic test for NP-C [1, 3], but it was not applied in our patient due to technical difficulties. Genetic testing has increasingly been used in the detection of NP-C over the last decade, particularly among patients with nonspecific, albeit suggestive, clinical symptoms. In this case, *NPC1* mutation analysis was only applied after the appearance of neurological disorders, and it identified known mutations allowing a full confirmation of the diagnosis.

NP-C pathognomonic symptoms such as vertical supranuclear ophthalmoparesis, and cerebellar signs such as ataxia, dysarthria, and dystonia, only became evident 2 years ago (when our patient was 3-years old). The comprehensive characterization of the time of onset, nature, and severity of neurological signs is considered vital for diagnosing NP-C, but can be very challenging in children below 2 years of age [3]. A recently developed pediatric version of the NP-C suspicion index alongside plasma oxysterol testing might help in this respect, in future cases [5].

## Conclusions

A targeted treatment for neurological deterioration that has been reported to slow disease progression and improve patients’ quality of life is now available for NP-C [3]. In view of this case, we consider it important to examine all newborns and children who display unexplained cholestasis or isolated splenomegaly/hepatosplenomegaly during the first months of life for signs of possible NP-C.
